# A new treatment using low level laser therapy for dehiscence saphenectomy post myocardial revascularization in diabetic patients

**DOI:** 10.1186/1749-8090-8-S1-O121

**Published:** 2013-09-11

**Authors:** NC Pinto, PMA Pomerantzeff, MHC Pereira, F Jatene, MC Chavantes

**Affiliations:** 1 Cardiovascular Surgery Department, Heart Institute (InCor), Hospital das Clínicas da Faculdade de Medicina da Universidade de São Paulo, São Paulo, Brazil; 2 Anesthesiology Department, Heart Institute (InCor), Hospital das Clínicas da Faculdade de Medicina da Universidade de São Paulo, São Paulo, Brazil

## Background

In Brazil, the main cause of death is the coronary heart disease and the surgical treatment used is the Myocardial Revascularization (MR). Patients undergoing to MR through bypass saphenous vein have been developed dehiscence in 10%. Dehiscence of surgical incision through Bioestimulation treatment with Low Level Laser in patients that underwent to MR seems to be an unprecedented new treatment and less invasive technique in the medical armamentarium.

## Methods

It was analyzed 10 diabetic patients, mean age 65,3 years that after surgery of MR evaluated for dehiscence surgical saphenectomy in lower member as well as edema and pain. Diode Laser C.W. (685 nm wavelength) with Power= 25 mW, Time= 30 s, Fluency= 4J/cm2 was applied, punctually surrounding surgical wound’s sore every 2 cm.

## Results

It revealed early reduction of the pain, as well as improved the health appearance of granulated tissue, decrease inflammatory process, wound’s size and reduction fibrin since the very first application. In superficial wounds only 3 sessions was required, while in the extensive wounds 8-10 applications was necessary.

## Conclusion

LLLT were effective in tissue repair, closing the saphenectomy dehiscence with a substantial improvement of patient’s quality of life, that benefits patients and Institutions, with cost-effectiveness, beyond denoted to be a less invasive treatment.

